# The effect of My Health Record use in the emergency department on clinician-assessed patient care: results from a survey

**DOI:** 10.1186/s12911-022-01920-8

**Published:** 2022-07-05

**Authors:** Alexandra Mullins, Renee O’Donnell, Heather Morris, Michael Ben-Meir, Kostas Hatzikiriakidis, Lisa Brichko, Helen Skouteris

**Affiliations:** 1 School Public Health and Preventative Medicine, Monash University, 553 St Kilda Road, Melbourne, VIC 3004 Australia; 2 Warwick Business School, University of Warwick, Scarman Rd, Coventry, CV4 7AL UK; 3 Cabrini Health, 181-183 Wattletree Rd, Malvern, VIC 3144 Australia; 4 Austin Health, Heidelberg, VIC Australia; 5 Alfred Emergency and Trauma Centre, Melbourne, VIC 3004 Australia; 6 Department of Critical Care, Melbourne Medical School, Melbourne University, Melbourne, VIC 3004 Australia

**Keywords:** Electronic health record, My Health Record, Emergency department, Personally Controlled Electronic Health Record, Medical systems

## Abstract

**Background:**

The emergency department has been a major focus for the implementation of Australia’s national electronic health record, known as My Health Record. However, the association between use of My Health Record in the emergency department setting and patient care is largely unknown. The aim of this study was to explore the perspectives of emergency department clinicians regarding My Health Record use frequency, the benefits of My Health Record use (with a focus on patient care) and the barriers to use.

**Methods:**

All 393 nursing, pharmacy, physician and allied health staff employed within the emergency department at a tertiary metropolitan public hospital in Melbourne were invited to participate in a web-based survey, between 1 May 2021 and 1 December 2021, during the height of the Delta and Omicron Covid-19 outbreaks in Victoria, Australia.

**Results:**

Overall, the survey response rate was 18% (70/393). Approximately half of the sample indicated My Health Record use in the emergency department (n = 39, 56%, confidence interval [CI] 43–68%). The results showed that users typically only engaged with My Health Record less than once per shift (n = 15, 39%, CI 23–55%). Just over half (n = 19/39, 54%, CI 32–65%) of all participants who use My Health Record agreed they could remember a time when My Health Record had been critical to the care of a patient. Overall, clinicians indicated the biggest barrier preventing their use of My Health Record is that they forget to utilise the system.

**Conclusion:**

The results suggest that My Health Record has not been adopted as routine practice in the emergency department, by the majority of participants. Close to half of self-identified users of My Health Record do not associate use as being critical to patient care. Instead, My Health Record may only be used in scenarios that clinicians perceive will yield the greatest benefit—which clinicians in this paper suggest is patients with chronic and complex conditions. Further research that explores the predictors to use and consumers most likely to benefit from use is recommended—and strategies to socialise this knowledge and educate clinicians is desperately required.

**Supplementary Information:**

The online version contains supplementary material available at 10.1186/s12911-022-01920-8.

## Background

To date the Australian government has invested close to $2 billion in the development and implementation of an electronic health record (EHR), known as My Health Record (MHR) [1]. MHR is a national EHR that was launched in 2012 as an opt-in model. In January 2019 MHR transitioned to an opt-out model, and as a result, approximately 90% of Australians now have a MHR [2]. Unlike an internal electronic medical record that is limited to one healthcare service, MHR is nationally available—via an online portal—to authorised providers and may contain information such as medications, immunisations history, pathology reports or specialist letters. In addition, MHR is a personally controlled EHR, therefore it is the consumers choice who can view and contribute information to their record. The emergency department (ED) has been a major focus for MHR implementation, given access to medical information in the ED can mean the difference between an intervention that is life-saving or life-threatening [3–5]. Notably, simply implementing the MHR system does not guarantee improved patient care and/or outcomes for patients who present to the ED [6].

Since the adoption of EHRs in hospitals, clinicians and academics have explored how they can be used to facilitate and improve patient care [7], in particular through instant access to patient data and improved information sharing. However, evidence regarding the impact of EHR use in hospitals on the quality of patient care is mixed [8, 9]. While the use of EHR systems has been associated with improved documentation of patient care, clinical processes [10], ambulatory care quality and cost and utilisation efficiencies [9]—EHR use has also been linked to challenges associated with increased documentation time for staff, clinician burnout and a reduction in the time clinicians have available to spend with patients [8, 11].

Much less research is available that explores the impact of EHR use in the time critical ED setting, on patient care [12]. The systematic review by Mullins, O’Donnell [12] captures only 23 studies between 2000 and 2019 that assess the impact of EHRs on healthcare outcomes. One study, by Ben-Assuli, Sagi [13], utilised simulation as a research method to explore the impact of a large scale interoperable EHR system in Israel,^1^ and concluded that EHR use in the ED leads to improved clinical decision quality and faster/more efficient decision-making. To the authors knowledge, only one study is available that explores the impact of use of Australia’s MHR in the ED, from the perspective of clinicians—the primary end users of the system [14]. Authors Mullins, Morris [15] expose (through a series of interviews and surveys) that some clinicians who use MHR report improved patient care and patient outcomes (including through reductions in exposure to nephrotoxic agents and improved diagnostic accuracy). Given the sizeable investment into MHR implementation and the long-awaited and anticipated benefits for ED staff and patients, the lack of knowledge regarding the impact of MHR use in the ED on patient care is of concern [16].

Understanding end users' perspectives towards MHR is vital to ensuring success [14]. As such, the overall objective of this study was to explore the perspectives of ED doctors, pharmacists, nurses and allied health staff on MHR use frequency, the benefits of MHR use (with a focus on patient care) and the barriers to MHR use.

## Methods

### Study setting

This study was conducted prospectively at Austin Health Emergency Department in Melbourne, Australia, which is a tertiary metropolitan public hospital that manages approximately 90,000 ED attendances per annum.^2^ Austin Health implemented MHR in 2016 and supports one-click access to MHR through an icon on each patient file.^3^

### Study design

This study was performed over an 8-month period from 1 May 2021 to 1 December 2021, and involved the distribution of a web-based survey to ED employees. Given this research was conducted during the COVID-19 pandemic, and EDs were experiencing unprecedented demand (resulting in increased ambulance arrivals, overcrowded waiting rooms and staff burnout [17]), the authors anticipated that recruitment of ED clinician’s may be restricted. Despite such challenges, the authors proceeded with the data collection, given MHR facilitates access to patient immunisation history (including COVID vaccine status)—insight which is anticipated to be highly sought after at this time.

**Fig. 1:**
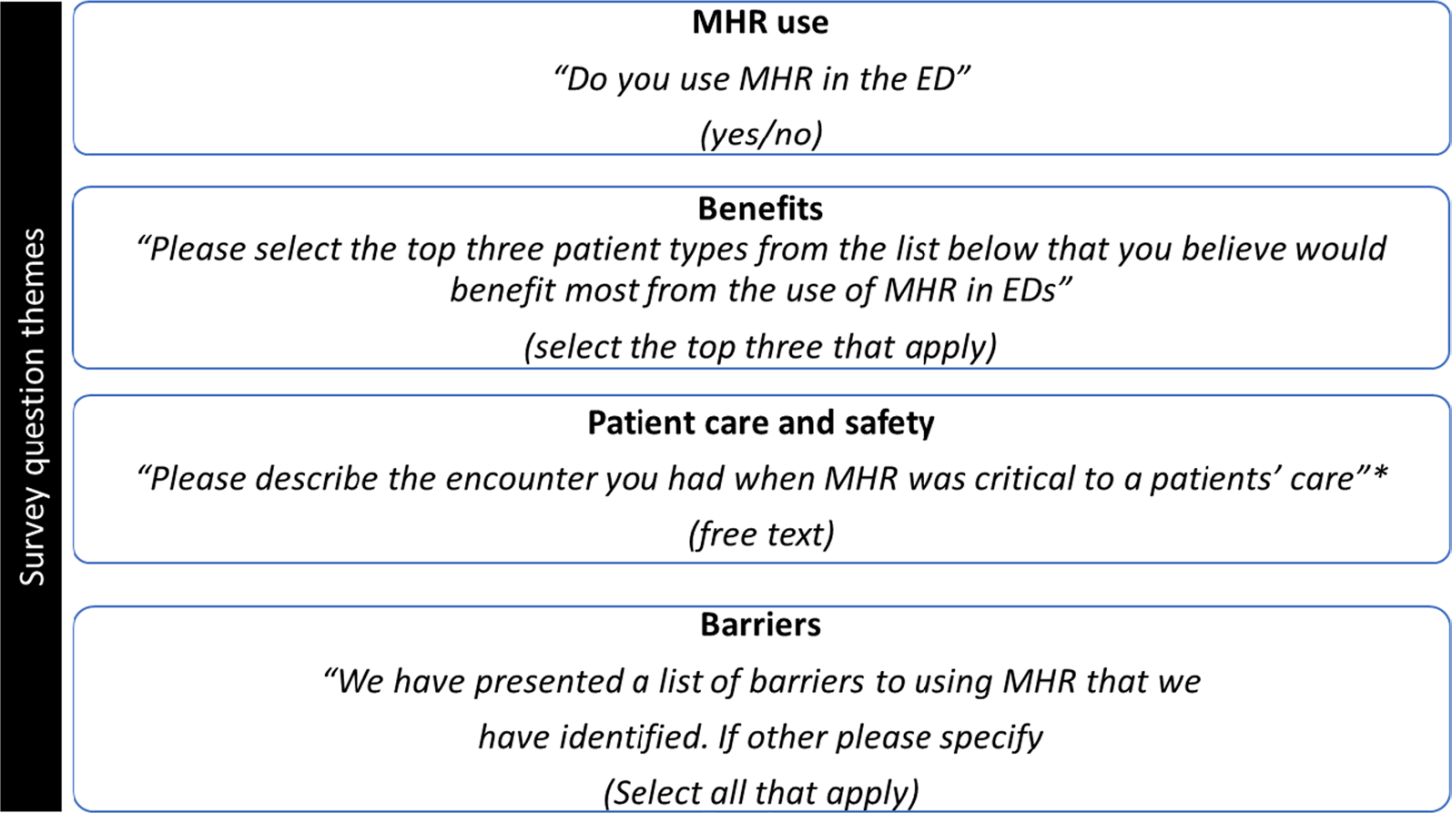
Survey themes and example questions. *It was only possible for a participant to complete this question if they agreed to using MHR. *MHR* My Health Record, *ED* emergency department

The 17-item survey (Additional file 1) was hosted on the Qualtrics online survey platform, guided by the survey instruments previously produced and utilised by authors Melvin, Saef [18] and Mullins, Morris [15] who explored ED clinicians’  opinions of large scale EHRs. To optimise the insights and quality of the research results generated from the survey, the survey was pilot tested (a total of four pilot surveys were completed by two clinicians and two medical informatics professionals) [19]. In addition to preliminary questions regarding participant demographics, the survey assessed several areas related to clinicians’ perspectives of MHR, including: use frequency; the barriers to use; and, the benefits associated with use (focusing on patient care and safety). In order to minimise response bias—in the form of socially desirable responding [20]—we purposefully asked staff about MHR use in a way that would not alert clinicians that use of MHR should be routine practice (see Fig. 1 for example survey questions). All survey items required either a multiple choice, yes/no or free text response, except for one question which required a response on a 5-point Likert scale (anchored at 1 = strongly disagree, 3 = undecided, 5 = strongly agree).

### Recruitment

A purposive convenience sampling method was used to sample all physician, pharmacy, nursing and allied health or administration staff employed within Austin Health ED (n = 127, n = 12, n = 246 and n = 8, respectively). In order to maximise the participant response rate, clinicians were invited to participate in the survey via their respective team meetings—directly by the lead researcher (who shared a link to the digital survey). Those who chose to participate within staff meetings were allocated up to five minutes to complete the survey within the meeting, without interruption. An invitation to participate in the survey was also distributed (to the previously mentioned groups, as many staff were not present at the staff meetings) via email. No participation incentives were offered as this survey was completed during paid-work hours.

### Statistical analysis

The sample was separated into two groups (self-identified users of MHR and non-users of MHR). Descriptive statistics were performed to compute frequency counts and percentages to compare across and within the two predefined groups, in addition to confidence intervals, using the Statistical Package for the Social Sciences (SPSS TM version 19.0; Chicago, IL, USA).

### Ethics

The protocol for the study was reviewed and approved as a Quality Improvement Project by the Austin Human Research Ethics Committee in May 2021 (QI project 40998). Participation in the study was on the basis of informed consent and the study was carried out in compliance with the Helsinki Declaration.

## Results

Overall, the survey response rate was 18% (70/393), which included n = 29 physicians, n = 7 pharmacists, n = 26 nurses and n = 8 allied health staff/other. The response rate was encouraging given the stress the workforce was under during the survey period (with respect to the COVID-19 pandemic [17]) and noting that surveying physician’s is reportedly challenging therefore a lower response rate than the general population is anticipated [21].

### Characteristics of MHR users

Over half (n = 39, 56%, 95% confidence interval [CI] 43–68%) of the participants in the sample reported they use MHR in the ED (see Table 1). The results indicate that pharmacists were the greatest users of MHR (n = 7, 100%, CI 59–100%). The group of participants who were between 30 and 39 years of age (n = 21, 70%, CI 51–85%) indicated greater MHR use than participants aged 18–29 or over 40. Overall, participants with less years of total ED experience (0–4 years) (n = 16, 67%, CI 45–84%) indicated greater MHR use than participants with a greater number of years of experience in the ED (5 + years). Participants with between 5–9 years worked in the ED at the study site (n = 9, 69%, CI 39–91%) indicated greater MHR use than participants with 0–4 years and over 10 years worked in the ED at the study site. Participants who worked more than 40 h per week in the ED (n = 12, 80%, CI 52–96%) indicated greater MHR use than those who worked between 8 and 40 h (n = 27, 49%, CI 35–63%).

**Table 1:** Characteristics of self-identified user’s vs non-users of My Health Record

Survey question	Response	Use MHR (n = 39)			Do not use MHR (n = 31)			Total	
		N	% Total^a^	% Total^b^	N	% Total^a^	% Total^b^	N	% Total
ED role	Doctor	20	68.97	51.28	9	31.03	29.03	29	41.43
	Pharmacist	7	100.00	17.95	0	0.00	0.00	7	10.00
	Nurse	8	30.77	20.51	18	69.23	58.06	26	37.14
	Administration	0	0.00	0.00	2	100.00	6.45	2	2.86
	Allied Health	1	50.00	2.56	1	50.00	3.23	2	2.86
	Care coordination team	3	75.00	7.69	1	25.00	3.23	4	5.71
	Total	39	56.00	100	31	45.00	100	70	100
Gender	Male	10	52.63	25.64	9	47.37	29.03	19	27.14
	Female	27	56.25	69.23	21	43.75	67.74	48	68.57
	Non-Binary/Gender Diverse	1	100.00	2.56	0	0.00	0.00	1	1.43
	Prefer not to say	1	50.00	2.56	1	50.00	3.23	2	2.86
	Total	39	55.71	100.00	31	44.29	100.00	70	100.00
Age	18–29 years	8	50.00	20.51	8	50.00	25.81	16	22.86
	30–39 years	21	70.00	53.85	9	30.00	29.03	30	42.86
	40–49 years	7	50.00	17.95	7	50.00	22.58	14	20.00
	50–59 years	3	30.00	7.69	7	70.00	22.58	10	14.29
	Total	39	55.71	100.00	31	44.29	100.00	70	100.00
Years of ED experience	0–4 years	16	66.67	41.03	8	33.33	25.81	24	34.29
	5–9 years	9	50.00	23.08	9	50.00	29.03	18	25.71
	10–19 years	11	57.89	28.21	8	42.11	25.81	19	27.14
	20–29 years	3	33.33	7.69	6	66.67	19.35	9	12.86
	Total	39	55.71	100.00	31	44.29	100.00	70	100.00
Years worked in the ED at the study site	0–4 years	22	56.41	56.41	17	43.59	54.84	39	55.71
	5–9 years	9	69.23	23.08	4	30.77	12.90	13	18.57
	10–19 years	7	58.33	17.95	5	41.67	16.13	12	17.14
	20–29 years	1	16.67	2.56	5	83.33	16.13	6	8.57
	Total	39	55.71	100.00	31	44.29	100.00	70	100.00
Hours worked per week in the ED	8–40 h	27	49.09	69.23	28	50.91	90.32	55	78.57
	More than 40 h	12	80.00	30.77	3	20.00	9.68	15	21.43
	Total	39	55.71	100.00	31	44.29	100.00	70	100.00

NB/GD: *non-binary/gender diverse; + prefer not to say; ^a^Total of all respondents; ^b^Total within user group (either user or non-user); *ED* emergency department

### Frequency of use and change in use over time

The results showed that just over a third of users indicated that they engaged with MHR less than once per shift (n = 15, 39%, CI 23–55%). A total of 11 clinicians (28%, CI 15–45%) who responded that they use MHR, indicated that they do so for every patient possible in the ED each shift. Interestingly, 67% (n = 26, CI 50–81%) of participants who indicated MHR use, reported that they use MHR more so now than they did in the prior 12 months.

### Barriers to use

Table 2 presents the relevant descriptive statistics for barriers to MHR use. The most common barriers reported by participants who identified as users were: patients do not request information to be uploaded into their record (n = 18, 53%, CI 35–70%); MHR is not user friendly (n = 15, 44%, CI 27–62%); MHR does not have the information required (n = 14, 41%, CI 25–59%); not enough healthcare providers use MHR (n = 14, 41%, CI 25–59%); and, forgetting to use MHR (n = 13, 38%, CI 22–57%). In contrast, the most common barriers reported by participants who identified as non-users were: forgetting to use MHR (n = 13, 45%, CI 26–64%); not knowing how to use MHR (n = 10, 35%, CI 18–54%); and, the perception that no one else in the ED used MHR (n = 8, 28%, CI 13–47%). Overall, the most frequently reported barrier to use that users and non-users reported was that they forget to use MHR (n = 26, 41%, CI 29–64%).

**Table 2:** Barriers to My Health Record (MHR) use

Barriers to MHR use	Use MHR (n = 34 respondents)			Do not use MHR (n = 29 respondents)			Total (n = 63 respondents)	
	N	% Total^a^	% Total^b^	N	% Total^a^	% Total^b^	N	%
No time to use MHR	5	50.00	14.71	5	50.00	17.24	10	15.87
MHR does not have the information required	14	73.68	41.18	5	26.32	17.24	19	30.16
Information within MHR can be found a quicker way	3	60.00	8.82	2	40.00	6.90	5	7.94
Unsure how to use MHR	5	33.33	14.71	10	66.67	34.48	15	23.81
No ED staff appear to use MHR	4	33.33	11.76	8	66.67	27.59	12	19.05
Forget to use MHR	13	50.00	38.24	13	50.00	44.83	26	41.27
Help and support services are not useful	5	71.43	14.71	2	28.57	6.90	7	11.11
Patient’s do not request information to be uploaded into their record	18	85.71	52.94	3	14.29	10.34	21	33.33
Information in MHR is not accurate or up to date	12	70.59	35.29	5	29.41	17.24	17	26.98
Using MHR impacts workflow	4	80.00	11.76	1	20.00	3.45	5	7.94
Concerns about privacy and security of information in MHR	2	40.00	5.88	3	60.00	10.34	5	7.94
MHR is not user friendly	15	93.75	44.12	1	6.25	3.45	16	25.40
Poor internet connection in the ED/a long waiting time to load	5	83.33	14.71	1	16.67	3.45	6	9.52
Not enough healthcare providers use MHR	14	73.68	41.18	5	26.32	17.24	19	30.16
No barriers are applicable to me	1	20.00	2.94	4	80.00	13.79	5	7.94
Other*								
Delay in the upload of information to MHR	1	100.00	2.94	0	0.00	0.00	1	1.59
Administration have not entered Medicare number required for access	1	100.00	2.94	0	0.00	0.00	1	1.59
No access due to role (e.g., clerk)	0	0.00	0.00	1	100.00	3.45	1	1.59
Patient’s record is locked	1	100.00	2.94	0	0.00	0.00	1	1.59
Total	34	53.97	100.00	29	46.03	100.00	63	100.00

^*^Free text responses provided when other was selected; ^a^total of all respondents; ^b^total within user group (either user or non-user). Please note, non-users were grouped according to current use, therefore they may have previously used MHR

### Patient safety and quality of care

The patient safety and quality of care benefits associated with MHR use are presented in Table 3. Just over half (n = 19/39, 54%, CI 32–65%) of all participants who use MHR agreed they could remember a time when MHR had been critical to the care of a patient—of this subgroup, the majority (n = 17/19, 89%, CI 67–99%) provided an example of how it was critical to the care of the patient (via a free text response box). A common example reported was that MHR provided access to the patients’ medical or medication history when the patient could not communicate, omitted information or did not know (i.e. forgot). Examples demonstrated how MHR resulted in a change in the decision made (resulting in admission); more efficient decision making; improved diagnosis accuracy; avoided duplication of services; and/or, the avoidance of an adverse drug event).

**Table 3:** Patient safety and quality of care benefits associated with My Health Record (MHR) use (responses from a subsample of users only; n = 31)

Survey question	N^a^	%
Can you remember a time MHR has been critical to a patients' care?	19	54.29
MHR was critical because it provided access to the patient’s medical or medication history when*	17	89.47
The patient was unconscious and/or couldn’t communicate	8	47.06
The patient omitted information or didn’t know	5	29.41
Other	4	23.53
Benefits of using MHR	36	100
Efficiencies for staff	33	91.67
Efficiencies for patients	31	86.11
Cost savings for the hospital	13	36.11
Improved patient outcomes	26	72.22
Improved patient care	29	80.56
Access to information critical for patient safety	35	97.22
Influences decision making	29	80.56
Improves confidence in decision making	24	66.67
Decreases the time spent chasing information from other health services or professionals	31	86.11
Decreases the time spent communicating information to other health services or professionals	28	77.78
Provides clinical information that clinician’s do not normally have access to	27	75.00
Substitutes how clinician’s currently retrieve supplementary clinical information	19	52.78
Prevents staff from ordering a duplicate diagnostic test	21	58.33
Improved communication between services_	1	2.78
Access to information after hours_	1	2.78
Greater coordination of care_	1	2.78
Other	2	33.33
Patient types that benefit the most from MHR use^b^	36	100.00
Chronic and complex care	28	77.78
Indigenous	4	11.11
Inter-regional or interstate	10	27.78
Mental health	9	25.00
Culturally and linguistically diverse	20	55.56
Paediatric	1	2.78
Residential aged care	10	27.78
Unconscious	21	58.33
Other	1	2.78

^a^N (%) = Proportion of clinicians who agree with the statement

^b^Multiple choice question—Top 3 patient types only were selected

^*^17 of the 19 clinicians who responded that ‘yes’ to “can you remember a time when MHR was critical to patient care” described an encounter when MHR was critical to a patients’ care_Free text responses coded where possible when other was selected

The benefits of MHR were also explored via a multiple-choice question, where participants (only those who indicated MHR use) could select all benefits that they associate with their use of MHR in the ED. Access to information critical to patient safety was the highest rated benefit (n = 35, 97%, CI 86–100%), closely followed by efficiencies for staff (n = 33, 92%, CI 78–98%). Finally, well over half of all users report that MHR benefits patients who have chronic and complex care issues (n = 28, 78%, CI 61–90%), are unconscious (n = 21, 58%, CI 41–75%) or are culturally and linguistically diverse (n = 20, 56%, CI 38–72%).

## Discussion

Pharmacists, physicians, nurses and allied health staff from one Australian public hospital ED were surveyed to explore their perspectives of MHR use frequency, the benefits associated with MHR use (with a focus on patient care) and the barriers to use. Approximately half of the sample indicated they use MHR, however use typically involved engaging with MHR less than once per shift. Interestingly, the main barrier that clinicians report that impacts their use of MHR, is that they forget to use MHR. Users of MHR also indicate patients lack of MHR use/failure to request information is uploaded, is also a major barrier to use. Overall, a key result revealed through this research was that just over half of all participants in this study who indicated MHR use, could remember a time when MHR was critical to patient care. Finally, participants in this study suggest the patients most likely to benefit from MHR use are those with chronic and complex care issues.

Roughly half of the participants in this study indicate they use MHR in the ED. Yet, only 22% of these ‘users’ indicate they access the MHR for every patient possible in the ED. While this result is in line with access rates of other large scale EHRs (where use typically occurs in < 20% of patient presentations to the ED) [9, 12]), the findings suggests that routine practice of MHR use has not yet been achieved and that clinicians may only use MHR in scenarios they perceive will yield the biggest benefit for the MHR log-on [22]. Given mandating usage of alternative information sources has been described as a “prescription for inciting resistance” [23], further research is required that explores if the benefits outweigh the cost (i.e. with respect to patient outcomes and efficiencies) of using MHR for all patients, versus for selected patients only.

Only just over half of all participants in this study, who use MHR, indicate that MHR is critical to patient care. This finding deserves considerable attention, given a number of the major benefits associated with MHR use, proposed by the Australian Digital Health Agency,^4^ focus on improved patient outcomes [24]. In addition, this discovery may explain why the rates of use are so low amongst ED clinicians. While the results presented in this study align with proposed models of technology acceptance (that technology acceptance and use is influenced by the usability and usefulness of the technology [25]), there is insufficient research in this area that explores if the value attached to MHR is a reliable indicator of use, therefore more research is needed to identify what the motivators for use are. Such insight may help MHR implementers to address issues that arise at specific points in time during system implementation [26].

The results in our study suggest that use of MHR is strongly linked to clinicians remembering to use the system. This finding is important given models of technology acceptance that have been applied to EHR use historically, tend to focus on the link between the expected utility and usefulness of the technology [25, 27].This study highlights the importance of going beyond promoting utility and usefulness in implementation. Clinicians need  frequent reminders to use the system. For example, thoughtfully designed visual cues (prompts) that indicate the availability of information within MHR [28]—particularly for patients that are most likely to benefit when MHR is consulted.

Clinicians who use MHR highlight that patients lack of MHR use/failure to request information is uploaded into their record, is also a major barrier to their use. As MHR is consumer controlled, it is the consumers choice who can contribute information to their record, therefore if a consumer wants their medical history to be added to their MHR they must ask their general practitioner [20]. Consequently, there may be an opportunity to explore the broader system level barriers at play that could be contributing to MHR use in the community. Perhaps enhancing MHR use among patients will support to drive clinician use of MHR—as such, this is an area of research that demands further attention.

Another main finding that emerged from this study is that chronic and complex care patients are most likely to benefit from MHR use in the ED, as reported by clinicians. This result was anticipated (and aligns with previous literature conducted of EHRs in America [9]) as chronic conditions are known to require more information to minimise uncertainty through treatment and diagnosis [29]. This finding is significant given the anticipated potential MHR has to deliver efficiencies in the ED, and the need for more efficient care (driven by the current overcrowding crisis ED’s are reportedly facing [17], exacerbated by COVID-19).

The current study had a number of limitations. First, surveys are exposed to self-selection bias [30], therefore this survey may not be representative of the opinions of all ED clinicians—and may favour participation by MHR users versus MHR non users. Although this research captures valuable opinions of a nuanced and niche group of individuals, the barriers and benefits to use may not be all encompassing. Ideally to address the self-selection bias, the survey data could have been triangulated with qualitative interviews to provide a richer insight into clinicians’ perspectives (as exemplified in Mullins, Morris [15]), however given the COVID-19 crisis and associated demand experienced by ED staff at the time this research was conducted, this was not possible. Furthermore, clinicians in this study may have over-reported technology uptake and use [31]. In order to reduce this risk, this survey was carefully designed and piloted to ensure clinicians were encouraged to be open and honest in their responses. Notably, the views of patients were not captured in this research—and since MHR use among patients may be a contributing factor for clinician use—this may need to be explored in future research. Future research is also required that objectively explores the type of patients most likely to benefit from MHR use. Indeed, a specific focus on the impact of MHR use versus non-use on patient care, efficiencies, quality of care and cost in the busy ED environment also represents another notable area for future research. Finally, future research could also consider the risk generated by having an additional source of information available (in MHR) that is not accessed—and who is liable if the information within fails to be used to inform the patients care. This insight may also be helpful to policy makers.

## Conclusion

This novel paper sought to understand how Australia’s national, personally controlled EHR (MHR) is used in the ED, what the barriers to its use are, and what the perceived impact of use is on patient care in the busy, time-pressured, ED environment. The survey of clinicians conducted in this study suggests that MHR has not been adopted as routine practice in the ED, and that MHR may instead only be used in scenarios that clinicians perceive will yield the greatest benefit—provided clinicians actually remember to use the system. Further research that explores the predictors to use and patients most likely to benefit from MHR use is recommended.

## Supplementary Information

**Additional file 1.** The survey instrument employed in this research.

## Data Availability

The datasets used and/or analysed during the current study are available from the corresponding author on reasonable request.

## Abbreviations

MHR: My Health Record
ED: Emergency department
EHR: Electronic health record

## Footnotes

1 While Israel does not have a national EHR, the interoperable EHR included in this research is comparable to Australia’s MHR system as it is implemented in all hospitals in Israel and has over 3.5 million consumers (close to 40% of the total population).

2 Austin Health manages 500,000 patient care episodes annually—and encompasses a repatriation hospital, a rehabilitation centre, a cancer wellness centre and a research centre.

3 As of 2020, Austin health introduced an orientation competency specifically designed to educate staff, on commencement, regarding how to access MHR. No additional password verification requirements are required (once clinicians are already logged on to the local internal medical record system).

4 The Australian Digital Health Agency is the System operator of the MHR system and is a statutory authority in the form of a corporate Commonwealth entity.
