# Supplementary material for: The effect of My Health Record use in the emergency department on clinician-assessed patient care: results from a survey
Source: BMC Med Inform Decis Mak. 2022 Jul 5;22:178. doi: 10.1186/s12911-022-01920-8 (PMC9255536; doi:10.1186/s12911-022-01920-8)
Supplement: Supplementary file 1 — Additional file 1. The survey instrument employed in this research. [file 12911_2022_1920_MOESM1_ESM.docx]

APPENDIX A

Use of My Health Record in the ED: a survey

Start of Block: Default Question Block

**My Health Record in Emergency Departments Survey**

Q1
**Please complete this survey only once.**
 **Please confirm that you have read and understand the above and hereby consent to participate in this project.**

- I agree to participate (1)
- I do not agree to participate (2)

Skip To: End of Survey If Please complete this survey only once. Please confirm that you have read and understand the above... = I do not agree to participate

End of Block: Default Question Block

Start of Block: Block 1

Q_Demo **Demographic Questions**

Q2 Which Health Service do you work in?

________________________________________________________________

Q3 What is your role?

- Doctor (1)
- Pharmacist (2)
- Nurse (3)
- Other (please specify) (4) ________________________________________________

Q59 What is your gender

- Male (1)
- Female (2)
- Non-binary / gender diverse (3)
- My gender identity isn't listed. I identify as (4) ________________________________________________
- I prefer not to say (5)

Q54 Which of these age brackets are you in?

- 18-29 years (1)
- 30-39 years (2)
- 40-49 years (3)
- 50-59 years (4)
- 60+ years (5)

Q4 How many years of Emergency Department experience do you have?

- 0 - 4 years (1)
- 5 - 9 years (2)
- 10 - 19 years (3)
- 20 - 29 years (4)
- > 30 years (5)

Q5 How long have you worked at the Austin in the Emergency Department?

- 0 - 4 years (1)
- 5 - 9 years (2)
- 10 - 19 years (3)
- 20 - 29 years (4)
- > 30 years (5)

Q6 How many FTE hours do you do per week in the emergency department at Austin?

- Less than 1 hour per week (1)
- Between 8-40 hours per week (2)
- More than 40 hours per week (3)
- Between 1-8 hours per week (4)

End of Block: Block 1

Start of Block: Block 2

Q_Exp **Experience with My Health Record**

Q56 Do you use My Health Record in the Emergency Department?

- Yes (1)
- No (2)

Display This Question:

If Do you use My Health Record in the Emergency Department? = No

Q7 What is your experience with My Health Record? Select all that apply

- I have heard of it (1)
- I have seen it being used (2)
- I don't know what My Health Record is (6)

Display This Question:

If Do you use My Health Record in the Emergency Department? = Yes

Q7.1 You indicated that you have used My Health Record. Over the past year, do you (select the answer that applies)

- Use My Health Record more (1)
- Use My Health Record less (2)
- Use My Health Record the same amount (6)
- Not sure how use has changed (7)

Display This Question:

If You indicated that you have used My Health Record. Over the past year, do you (select the answer... = Use My Health Record more

Q7.1.1 You indicated that your use of My Health Record has changed in the last year. What factors have contributed to your change in use? (select all that apply)

- Greater awareness of My Health Record (1)
- Education on how to use My Health Record (2)
- Clinical champions (3)
- Knowing there is more information within My Health Record (4)
- Other (please specify) (5) ________________________________________________

Display This Question:

If Do you use My Health Record in the Emergency Department? = Yes

Q7.2 How often do you use My Health Record?

- less than once per shift (1)
- Once per shift (7)
- More than once per shift (8)
- Every patient possible each shift (9)

Display This Question:

If Do you use My Health Record in the Emergency Department? = No

Q7.3 You indicated that you do not use My Health Record. In the space provided, please briefly explain why.

________________________________________________________________

________________________________________________________________

________________________________________________________________

________________________________________________________________

________________________________________________________________

End of Block: Block 2

Start of Block: Block 10

Q_Aware **Awareness**

Q8

Please indicate the degree to which you agree with the following statements:

|  | Strongly Disagree (1) | Disagree (2) | Neutral (3) | Agree (4) | Strongly Agree (5) | Not Applicable (6) |
| --- | --- | --- | --- | --- | --- | --- |
| I know how to access My Health Record from my ED. (1) |  |  |  |  |  |  |
| I have heard or participated in conversations about My Health Record in the workplace. (2) |  |  |  |  |  |  |

End of Block: Block 10

Start of Block: Block 3

Q_Train **My Health Record Training**

Q9 What type of training or support did you receive in the last 12 months? Select all that apply

- Practical demonstrations showing how to access My Health Record (1)
- Information from peak bodies (2)
- Face-to-face training (3)
- Factsheets (4)
- My Health Record webinars (5)
- Discussions with colleagues (6)
- Other (please specify): (7) ________________________________________________
- I have not received any training (8)

Display This Question:

If What type of training or support did you receive in the last 12 months? Select all that apply != I have not received any training

Q55 Did the training received improve how often you access My Health Record?

- Yes (1)
- No (2)

Q10 Do you require more training to use My Health Record?

- Yes (1)
- No (2)

Display This Question:

If Do you require more training to use My Health Record? = Yes

Q10.1 Please elaborate on which aspects of using My Health Record you would like more training in.

________________________________________________________________

End of Block: Block 3

Start of Block: Block 5

Display This Question:

If Do you use My Health Record in the Emergency Department? = Yes

Q_Using **Using My Health Record**

Display This Question:

If Do you use My Health Record in the Emergency Department? = Yes

Q11 You indicated that you have used My Health Record. Please indicate the degree to which you agree with the following statements regarding your experience using My Health Record:

|  | Strongly Disagree (1) | Disagree (2) | Neutral (3) | Agree (4) | Strongly Agree (5) | Not Applicable (6) |
| --- | --- | --- | --- | --- | --- | --- |
| It is easy to access a patient's My Health Record. (1) |  |  |  |  |  |  |
| It is a quick process to find relevant clinical information once in My Health Record. (2) |  |  |  |  |  |  |
| Clinical information is presented in a clear way. (3) |  |  |  |  |  |  |
| Clinical information is relevant to my duties in the ED. (4) |  |  |  |  |  |  |
| I would be more likely to view my patient's My Health Record if they told me they had one. (5) |  |  |  |  |  |  |

Display This Question:

If Do you use My Health Record in the Emergency Department? = Yes

Q12 What suggestions do you have for how My Health Record clinical information is presented to improve ease of use? Please check all that apply.

- Apply a flag to patient records to indicate there is clinical information in My Health Record (1)
- Integrate My Health Record with the eMR/iMR interface (2)
- Supply best practice guidelines for My Health Record use (3)
- Comments or other suggestions: (4) ________________________________________________

End of Block: Block 5

Start of Block: Block 5

Q_Imp **Importance of Information in My Health Record**

Q13 Please rate the importance, of the following types of information being made available in a patients My Health Record, to you as a clinician:

|  | Not Important (1) | Slightly Important (2) | Moderately important (3) | Important (4) | Very Important (5) | Not Applicable (6) |
| --- | --- | --- | --- | --- | --- | --- |
| External radiologists' reports (1) |  |  |  |  |  |  |
| External radiologists' images (2) |  |  |  |  |  |  |
| External pathology results (3) |  |  |  |  |  |  |
| ECGs (4) |  |  |  |  |  |  |
| Advance care plans (5) |  |  |  |  |  |  |
| Patient's medication history (6) |  |  |  |  |  |  |
| Shared health summary (typically a patient's health summary from their GP) (7) |  |  |  |  |  |  |
| Hospital discharge summary (8) |  |  |  |  |  |  |

Q13.1 Please provide any comments or other suggestions you have regarding the importance of information included in My Health Record:

________________________________________________________________

End of Block: Block 5

Start of Block: Block 6

Q_Ben **Benefits of Using My Health Record**

Q14 What do you think are the benefits of using My Health Record? Please select all that apply.

- Efficiencies for staff (14)
- Efficiencies for patients (15)
- Cost savings for the Austin (16)
- Improved patient outcomes (17)
- Improved patient care (18)
- Provides access to information that is critical to patient safety (30)
- Influences clinical decision making (19)
- Improves confidence in clinical decision making (20)
- Decreases the time spent chasing information from other health services or professionals (23)
- Decreases the time spent communicating information to other health services or professionals (24)
- Provides clinical information that we do not normally have access to (25)
- Substitutes how we currently retrieve supplementary clinical information (26)
- Prevents staff from ordering a duplicate diagnostic test (27)
- There are none (13)
- Other (please specify) (29) ________________________________________________

Q15 Can you remember a time when My Health Record has been critical to a patients' care?

- Yes (1)
- No (2)

Display This Question:

If Can you remember a time when My Health Record has been critical to a patients' care? = Yes

Q15.1 Please describe the encounter you had when My Health Record was critical to a patients' care.

________________________________________________________________

End of Block: Block 6

Start of Block: Block 9

Q_Patient **Patient Types**

| 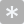 |
| --- |

Q16 Please select the **top three** patient types from the list below that you believe would benefit the most from the use of My Health Record in emergency departments:

- Chronic and complex care (1)
- Indigenous (2)
- Inter-regional or interstate (3)
- Mental health (4)
- Culturally and linguistically diverse (CALD) (5)
- Paediatrics (6)
- Residential Aged Care Facility (nursing home) (7)
- Unconscious (8)
- Other (please specify) (9) ________________________________________________

End of Block: Block 9

Start of Block: Block 7

Q_Barr **Barriers to Using My Health Record**

Q17 Below, we have presented a list of barriers to using My Health Record that we have identified. Please select all that apply to you:

- I don't have time to use My Health Record (1)
- My Health Record does not have the information I am looking for (2)
- I can find the information quicker another way (3)
- I don't know how to use My Health Record (4)
- No one around me in the ED uses My Health Record (5)
- I forget to use My Health Record (6)
- Help and support services for My Health Record are not useful (7)
- My patients do not use My Health Record (8)
- Patient information on My Health Record is not accurate or up to date (12)
- Using My Health Record impacts my workflow (13)
- I am concerned about the privacy and security of personal information in My Health Record (14)
- My Health Record is not user friendly (16)
- Poor internet connection prevents me from taking advantage of My Health Record (17)
- Not enough healthcare providers use My Health Record (18)
- Other (please specify) (19) ________________________________________________
- There are no barriers that are applicable to me (20)

End of Block: Block 7

Start of Block: Block 8

Q_Other **Would you like to add anything else about My Health Record?**

**If not, please click submit now**

________________________________________________________________

________________________________________________________________

________________________________________________________________

________________________________________________________________

________________________________________________________________

End of Block: Block 8
